# Draft genome sequence of the fungal biocontrol agent, *Bacillus velezensis* Kos

**DOI:** 10.1128/mra.00575-24

**Published:** 2024-08-27

**Authors:** Joy Clarke, Dejana Kosanovic, Kevin Kavanagh, Helen Grogan, David A. Fitzpatrick

**Affiliations:** 1 Department of Biology, Maynooth University, Maynooth, Ireland; 2 Horticulture Development Department, Teagasc, Dublin, Ireland; University of Guelph, Guelph, Ontario, Canada

**Keywords:** biocontrol agent, integrated pest management, *Bacillus velezensis* Kos, genomes

## Abstract

Here, we report the draft genome sequence of *Bacillus velezensis* strain Kos, isolated from casing soil used during *Agaricus bisporus* cultivation in Dublin, Ireland. *B. velezensis* Kos exhibits a suppressive ability toward *Cladobotryum mycophilum*, *Trichoderma aggressivum*, and *Lecanicillium fungicola*, which are common threats to *A. bisporus* production, cultivation, and quality.

## ANNOUNCEMENT

Several fungal pathogens pose a significant threat to the commercially important white mushroom, *Agaricus bisporus* (1). Historically, chemical fungicides have been used to prevent yield reductions and disease outbreaks. Due to environmental/health considerations, there is now pressure to reduce fungicide use (2). The future of mushroom disease treatment will depend upon integrated pest management, including the use of biological control agents (BCAs) (3). Here we report the draft genome sequence of the novel strain *Bacillus velezensis* Kos, which has been shown to have potential as a BCA for mushroom disease (4–6).

The Kos strain was originally isolated during *A. bisporus* cultivation in Dublin, Ireland (global positioning system coordinates 53.38 N 6.33 W″), and taxonomical identification showed that Kos is *Bacillus velezensis* (6), which we further confirmed using the average nucleotide identity (ANI) (see below). *B. velezensis* Kos plate cultures were grown on nutrient agar (Thermo Scientific Oxoid) at 30°C for 24 h. A loopful of culture from plate cultures was added to 50-mL nutrient broth (Thermo Scientific Oxoid) and grown for 24 h at 30°C, 120 rpm. Genomic DNA was extracted using the Quick-DNA Fungal/Bacterial Miniprep Kit (Zymo Research). The genome was sequenced by Novogene Co. Ltd., with the DNA library prepared using the Novogene NGS DNA library prep set in which the DNA was randomly sheared, end repaired, A-tailed, and then ligated with Illumina adaptors. These sequences were amplified using PCR, and DNA of 350 bp was selected, purified, and sequenced using 150-bp Illumina paired-end sequencing on the Illumina NovaSeq platform. Reads with adapters and low quality were trimmed using Skewer (v.0.2.2) (7).

In total 12,619,076 high-quality paired-end reads were obtained and initially assembled and then annotated using National Center for Biotechnology Information’s Read Assembly and Annotation Pipeline Tool with default settings (rapt-45639894). RAPT utilizes the SKESA (v.2.5.1) genome assembler (8), the ANI tool (9) to assign taxonomy and the Prokaryotic Genome Annotation Pipeline (build6771) (10) to functionally annotate the assembly. Genome quality and potential contamination are also assessed using CheckM (v.2015-01-16) (11). In total, the assembly size is 4,194,762 nucleotides in length with a GC content of 45.8%. The N50 and L50 scores are 573,424 and 3, respectively. CheckM showed 98.82% genome completeness and 0% contamination. The longest contig is 1,085,863 nucleotides, and there are 30 contigs in total. The ANI with its closest strain, *B. velezensis* NRRL B-41580 (12), was 98.264%. A total of 4,248 genes were predicted, including 4,066 protein-coding genes, 99 RNA genes (12 rRNAs, 82 tRNA, and 5 noncoding RNA genes), and 83 pseudogenes. The potential production of secondary metabolites by Kos was analyzed using the antiSMASH tool (v.7.0) with the default setting (13). Genomic clusters with the potential for the biosynthesis of antimicrobial secondary metabolites were predicted. These clusters involve genes encoding surfactin, subtilin, bacillibactin, bacilysin, fengycin, bacillaene, and macrolactin.

The draft genome sequence of *B. velezensis* Kos will help uncover the molecular mechanisms of pathogen suppression and increase its applications in the mushroom industry.

## Data Availability

This Whole Genome Shotgun project has been deposited at DDBJ/ENA/GenBank under accession number JBDOQF000000000. The version described in this paper is version JBDOQF010000000. The raw Illumina reads are available at ENA/SRA under accession number SRX24592991.
